# E-cadherin dynamics is regulated by galectin-7 at epithelial cell surface

**DOI:** 10.1038/s41598-017-17332-y

**Published:** 2017-12-06

**Authors:** Tamara Advedissian, Véronique Proux-Gillardeaux, Rachel Nkosi, Grégoire Peyret, Thao Nguyen, Françoise Poirier, Mireille Viguier, Frédérique Deshayes

**Affiliations:** 1 Team Morphogenesis, Homeostasis and Pathologies, University Paris Diderot, Sorbonne Paris Cité, CNRS UMR 7592, Institut Jacques Monod, 15 Rue Hélène Brion, 75013 Paris, France; 2 Team Membrane Traffic in Health & Disease, University Paris Diderot, Sorbonne Paris Cité, CNRS UMR 7592, Institut Jacques Monod, 15 Rue Hélène Brion, 75013 Paris, France; 3 Team Cell Adhesion and Mechanics, University Paris Diderot, Sorbonne Paris Cité, CNRS UMR 7592, Institut Jacques Monod, 15 Rue Hélène Brion, 75013 Paris, France

## Abstract

Re-epithelialisation of wounded epidermis is ensured by collective cell migration of keratinocytes. Efficient collective migration requires the maintenance of intercellular adhesion, notably through adherens junctions, to favour cell communication, support tension forces and coordinated movement . Galectin-7, a soluble lectin expressed in stratified epithelia, has been previously implicated in cell migration and intercellular adhesion. Here, we revealed a new function of galectin-7 in the control of directionality and collective behaviour in migrating keratinocytes. Consistently, we identified galectin-7 as a direct partner of E-cadherin, a key component of adherens junctions. Unexpectedly, this interaction does not require glycosylation motifs. Focusing on the underlying mechanisms, we showed that galectin-7 stabilizes E-cadherin at the plasma membrane, restraining its endocytosis. Interestingly, galectin-7 silencing decreases E-cadherin-mediated intercellular adhesion. Consequently, this study not only identifies a new stabilizer of adherens junctions but also emphasises the importance of the interplay between E-cadherin turnover and intercellular adhesion strength.

## Introduction

The skin is an essential organ that acts as a barrier thanks to its external layer, the epidermis, to protect the organism against environmental aggressions such as physical stress, chemical injury or infection. Hence, maintaining epidermal integrity throughout the lifetime of mammalian organisms is fundamental^1^. Following skin injury, epidermal keratinocytes located at the wound edge will migrate in a collective manner and proliferate to restore the epidermal protective barrier^2,3^. Collective cell migration is a type of cell displacement in which cells keep intercellular contacts while migrating^4^. During this process, intercellular adhesion complexes and in particular Adherens Junctions (AJs) play a crucial role to support cell-cell communication, to promote coordinated behaviour of the sheet of cells and to favour establishment of proper cell polarity^4–7^. AJs are cadherin-catenin based adhesion complexes that assemble at the cell surface where they maintain physical association between cells and mediate diverse intercellular signalling pathways such as proliferation or differentiation signalling pathways^8,9^. Trans-membrane cadherins become associated with the actin cytoskeleton by catenins, mainly α- and β-catenins, linking intercellular adhesion to the internal cytoskeleton. Through their role in cell-cell communication and their binding to the cytoskeleton, AJs promote the establishment of a multicellular network and favour coordination of the cell population as in collective cell migration during epithelial wound healing^10,11^.

In epithelial cells, E-cadherin-containing AJs play a crucial role in intercellular cohesion and communication, and in the modulation of the strength of intercellular adhesion^12^. At the cell surface, trans-membrane E-cadherin associates in a calcium-dependent and homophilic manner with E-cadherin molecules from adjacent epithelial cells^13^. In addition to these trans-interactions, the extracellular domain of E-cadherin forms cis-interactions with surrounding E-cadherin from the same cell^14^ and this clustering of E-cadherin favours anchoring of AJs to the actin cytoskeleton^15^. Different parameters regulate AJ-mediated adhesions such as protein level or complex dynamics at the plasma membrane. Indeed, E-cadherin undergoes constant turnover at the plasma membrane through endocytosis, recycling and sorting^12,16^. This constant renewal of E-cadherin in mature AJs is crucial during remodelling events^13^ but also in stationary epithelia to maintain intercellular contacts and support rapid adaptation to perturbations^12,17–19^. Depending on the regulators involved and the cell types, E-cadherin can travel through different endocytic pathways such as clathrin-dependent endocytosis, caveolin-mediated endocytosis or macropinocytosis^20–22^. Different proteins have been found to regulate E-cadherin stability at the plasma membrane including β-catenin, p120-catenin or tyrosine kinase receptors. However, how AJ dynamics is finely regulated still remains elusive and efforts are made to identify new stabilizing molecules and modulators of intercellular adhesion^13^.

Galectins are a family of small soluble lectins that share a conserved Carbohydrate Recognition Domain (CRD) and a common affinity for β-galactosides containing sugars^23^. They are located inside the cell, in the cytoplasm or in the nucleus, but also outside the cell. Galectins are secreted through an unconventional secretory pathway^24^. These proteins have been shown to participate in multiple processes including cell-cell interaction, intracellular trafficking, apoptosis and inflammatory responses^25^.

Galectin-7 is a lectin with a single CRD that has the ability to form homodimers^26,27^. Its expression is restricted to pluristratified epithelia such as the epidermis^28,29^. Galectin-7 null mice exhibit homeostasis defects under stress conditions. As an illustration, it was reported previously that this galectin is involved in the re-epithelialization process during skin and corneal wound healing^30–32^ and in the response to UV irradiation^31,32^. However, the function of galectin-7 in collective cell migration still remains to be elucidated.

Furthermore, our group demonstrated that both galectin-7 null mice (Gal7^-/-^) and galectin-7 overexpressing mice exhibit delayed wound healing and altered epidermal cohesion with the presence of intercellular spaces as visualized by ultrastructural imaging^31,32^. Interestingly, similar adhesion defects in the epidermis have been observed after conditional targeting of either E-cadherin^33,34^ or α-catenin^35^, two AJ proteins. Bearing in mind the importance of AJ-mediated adhesion during collective cell migration and the defects in cell-cell adhesion associated with the absence of galectin-7, we considered a potential interaction between galectin-7 and intercellular adhesion components, and aimed to decipher *in vitro* the underlying mechanisms.

In this study, we documented the involvement of galectin-7 in collective cell migration and unveil a new function of this galectin in the regulation of the collective behaviour during epithelial migration. Dissecting the causal mechanisms, we identified galectin-7 as a direct partner of the AJ protein E-cadherin, a central protein in cell coordination. By focusing on AJ dynamics, we showed that galectin-7 was a stabilizer of E-cadherin at the plasma membrane. Interestingly, an increase in E-cadherin turnover at the plasma membrane due to galectin-7 depletion is correlated with reduced AJ mediated adhesion strength, providing a new illustration of the importance of the regulation of AJ dynamics to maintain proper intercellular adhesion.

## Results

### Galectin-7 depletion influences collective cell movement

To decipher the role of galectin-7 in collective cell migration, we used HaCaT cells, an immortalized keratinocyte human cell line, which form a collectively migrating monolayer in response to *in vitro* wounding. We generated two stable HaCaT clones with a highly reduced expression of galectin-7 thanks to the expression of an shRNA targeting galectin-7 mRNA (shGal7 clones, supplementary Figs S1a and S1b).

**Figure 1:**
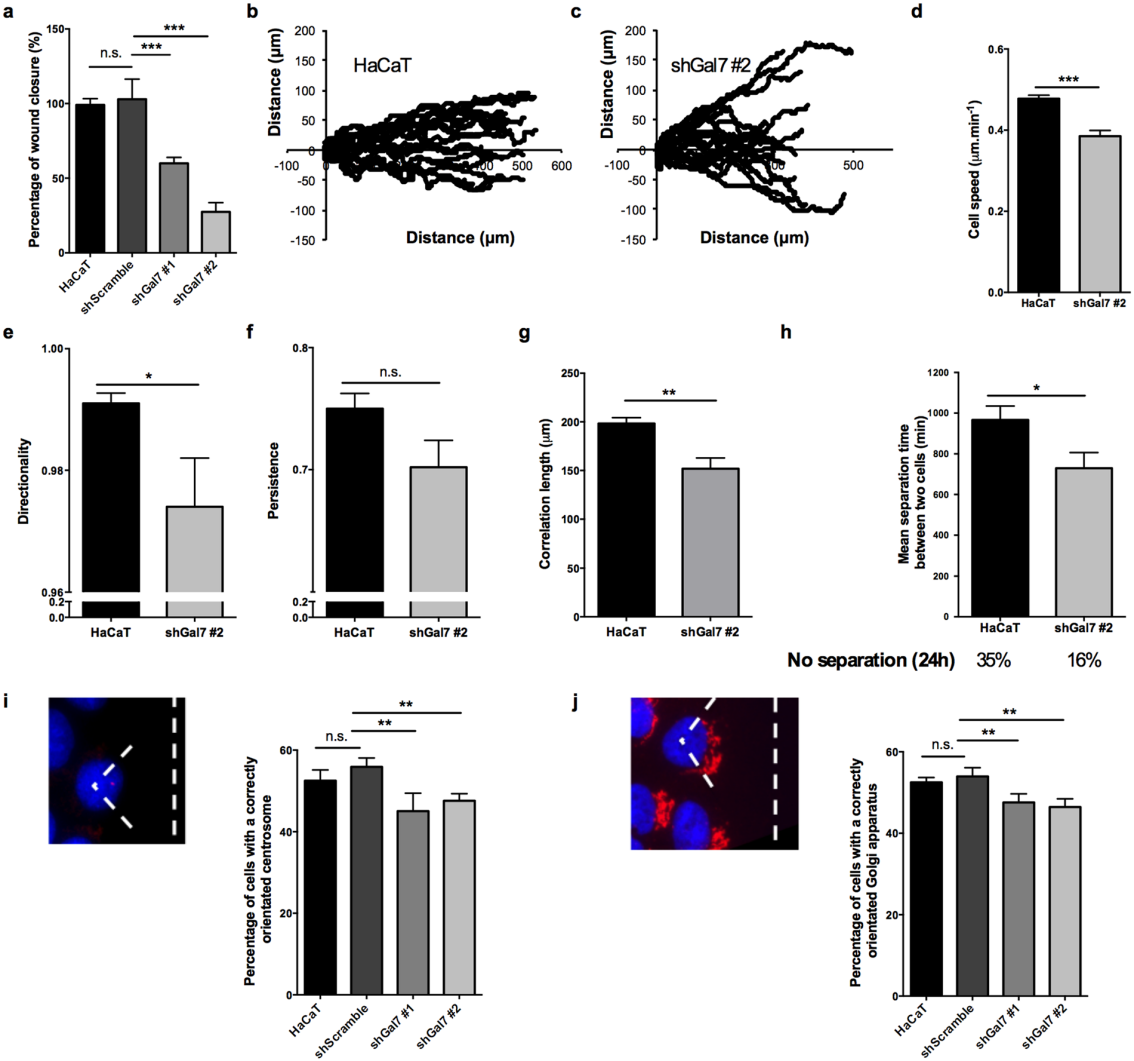
Galectin-7 downregulation impairs collective cell migration of HaCaT keratinocytes and reduce collective behaviour. (**a**) Percentage of wound closure normalized to HaCaT WT cells in a inserts removals wound healing experiment. Mean ± s.e.m. are represented (n = 5). (**b**) Single cell tracking of collectively migrating HaCaT cells at the front of the free edge during 24 h after insert removal. (**c**) Mean individual cell speed was extracted from cell trajectories. Mean ± s.e.m. are represented. (**d**) Effect of galectin-7 downregulation on directional movement of HaCaT cells at 18 h. Mean ± s.e.m. are represented. (**e**) Measurement of the persistence parameter extracted from trajectory curves at 18 h. Mean ± s.e.m. are represented. P-value = 0.069. (**f**) Correlation length measured in collectively migrating HaCaT or shGal7 #2 clone. Mean ± s.e.m. are represented. (**g**) Mean separation time between two neighbouring cells analysed manually from movies of migrating cells during 24 h. The proportion of cells that did not separate during this interval is written under the graph. Mean ± s.e.m. are represented. (**h**) Centrosome orientation in collectively migrating HaCaT cells. The proportion of front cells with centrosome located in the 90° angle facing the wound has been quantified. (**i**) Golgi apparatus orientation in collectively migrating HaCaT cells. The proportion of front cells with the majority of the Golgi apparatus located in the 120° angle facing the wound has been quantified. (**h**,**i**) For each graph, 4 independent experiments, with a total of at least 100 cells per experiment have been analyzed. Results shown are mean values ± s.d. P-values obtained from unpaired t-tests with Welch’s correction are represented. ***p < 0.001; **p < 0.01; *p < 0.05.

We first set up an *in vitro* woundhealing assay using insert removal technics to investigate the migratory potential of galectin-7 depleted clones. Sixteen hours after insert removal, the shGal7 clones exhibited a significant delay in wound healing of respectively 40% (shGal7 #1) and 70% (shGal7 #2) compared to control HaCaT cells (Fig. 1a), highlighting the involvement of galectin-7 in collective cell migration. Interestingly, the migration delay correlated with the level of galectin-7 silencing (supplementary Fig. S1b).

To better understand the migratory defects associated with galectin-7 depletion, we performed video-microscopy of migrating control HaCaT cells or shGal7 #2 clone, the clone with the lowest amount of galectin-7, and followed the trajectory of randomly chosen front cells (supplementary video 1 and 2). The cell trajectories obtained revealed that galectin-7 depleted cells displayed a less straightforward movement compared to control HaCaT cells (Fig. 1b). From these trajectories, the cell speed was measured showing that the silencing of galectin-7 significantly reduced individual front cell speed (Fig. 1c). The directionality value, which represents the ability of the cells to migrate in the direction of the wound, was significantly lower in the shGal7 #2 clone (Fig. 1d). Furthermore, the persistence parameter, which reflects the ability of the cells to maintain their orientation in a given direction, was reduced in the shGal7 #2 clone but this tendency did not reach significance (Fig. 1e, p-value = 0.069).

To assess more precisely the collective behaviour of the cells after galectin-7 silencing, we measured the velocity correlation length, which is the distance over which cell movements does not show co-variation of the direction of velocity vector^36^. ShGal7 #2 clone displayed a decrease in the correlation length of about 46 μm compared to control HaCaT cells (Fig. 1f). In addition, we evaluated the mean separation time between migrating cells that were neighbours at t = 0 h, when the insert was removed. Interestingly, galectin-7 depleted cells exhibited a 4 h lower separation time, indicating that they exchanged neighbours faster (Fig. 1g). In agreement with this result, only 16% of shGal7 #2 cells remained in contact during the 24 h covered by the movie whereas this proportion was about 35% in control HaCaT cells. These observations indicate that the delay in wound healing observed after galectin-7 knockdown may be due in part to a reduced migration cell speed but also to a decreased coordination between cells.

A decrease in directionality is often associated with a reduced capacity of cells to establish front-to-rear polarity. Thus, to analyse cell polarity at the migration front after galectin-7 silencing, we evaluated the positioning of the centrosome and the Golgi apparatus in these cells^37^. Indeed, during collective cell migration, centrosome and Golgi apparatus localise in front of the nucleus in the direction of the wound^4^. As shown in Fig. 1h and 1i, galectin-7 downregulation is associated with a decrease in the proportion of correctly orientated cells, indicating a defect in front-to-rear polarity. Hence, these data show that galectin-7 depletion limits collective behaviour during migration and impairs the establishment of cell polarity.

### Galectin-7 interacts with AJ components in a human keratinocyte cell line

**Figure 2:**
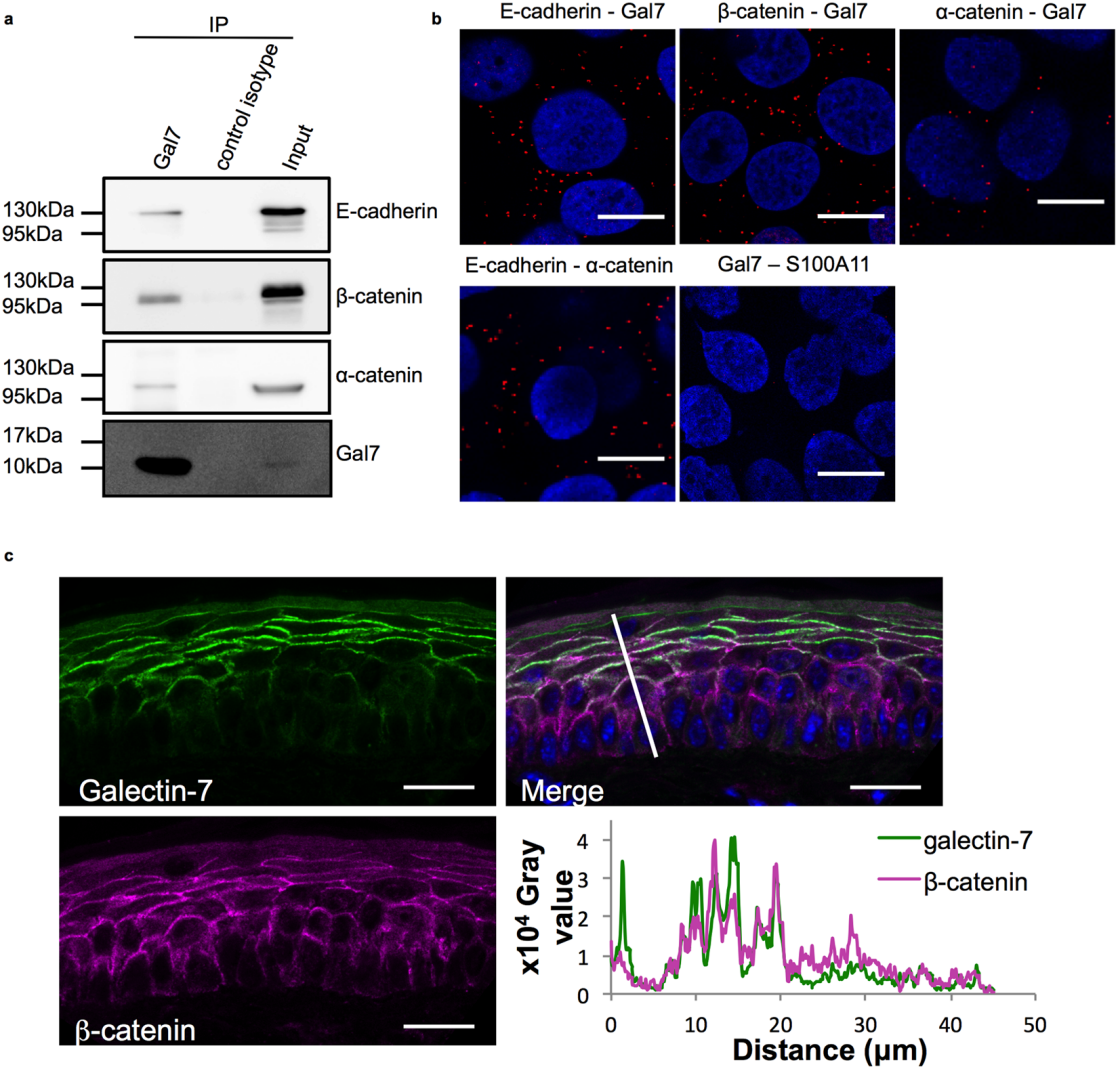
Galectin-7 interacts and colocalizes with adherens junction components. (**a**) Co-immunoprecipitation experiments indicate that galectin-7 is a partner of E-cadherin, α-catenin and β-catenin. Images shown are representative images taken from distinct western blots. (**b**) Confocal images of Proximity Ligation Assays confirming that galectin-7 is in close proximity with distinct AJ components in the cell context. E-cadherin – α-catenin and galectin-7 – S100A11 pairs were used as respectively positive and negative controls. Gal7 = Galectin-7. Scale bars = 15 μm. (**c**) Representative immunostaining of galectin-7 (green) and β-catenin (magenta) in WT mice tail epidermis showing co-localisation of these two proteins. Scale bar = 20 μm. Intensity values measured along the white line were plotted in function of the distance from the base of the epidermis. Pearson’s r coefficient for galectin-7 / β-catenin staining = 0.63 ± 0.03, n = 6.

To better understand how galectin-7 might influence collective cell migration, we aimed at identifying galectin-7 partners whose function is relevant to collective migration. Previous studies performed in our team indicate that galectin-7 interacts with the AJ protein E-cadherin^32^. Because AJs play a central role in collective behaviour and establishment of polarity^4,38,39^, we focused our analysis on this candidate complex. Co-immunoprecipitation experiments indeed revealed that galectin-7 co-precipitated the AJ proteins E-cadherin, α-catenin and β-catenin (Fig. 2a) and conversely that these AJ proteins precipitated galectin-7 (supplementary Figs S2a, S2b and S2c).

To ascertain that this association was effective in living cells, we performed a proximity ligation assay (PLA). This approach indicates if two proteins are in close proximity (less than 40 nm) with the appearance of red fluorescent dots at the location of their interaction. Like the E-cadherin/α-catenin positive control, galectin-7/β-catenin, galectin-7/α-catenin and galectin-7/E-cadherin pairs showed close association (Fig. 2b). As expected, the negative control staining of galectin-7 with S100A11, a calcium-binding protein localised in the cytoplasm of keratinocytes, exhibited only few disparate dots (Fig. 2b).

To explore *in vivo* the co-localisation of these molecules, immunostaining was conducted on tail skin biopsies in mice. Galectin-7 was found to co-localize with the AJ component β-catenin at cell-cell contacts in the upper layers of the mouse epidermis (Pearson’s r coefficient for galectin-7/β-catenin staining = 0.63 ± 0.03, n = 6, Fig. 2c).

Taken together, these results strongly suggest that galectin-7 is associated with the AJ complex containing E-cadherin, α-catenin and β-catenin and co-localizes with AJs at the cell contacts in mouse epidermis.

### Galectin-7 directly interacts with E-cadherin through the extracellular domain

**Figure 3:**
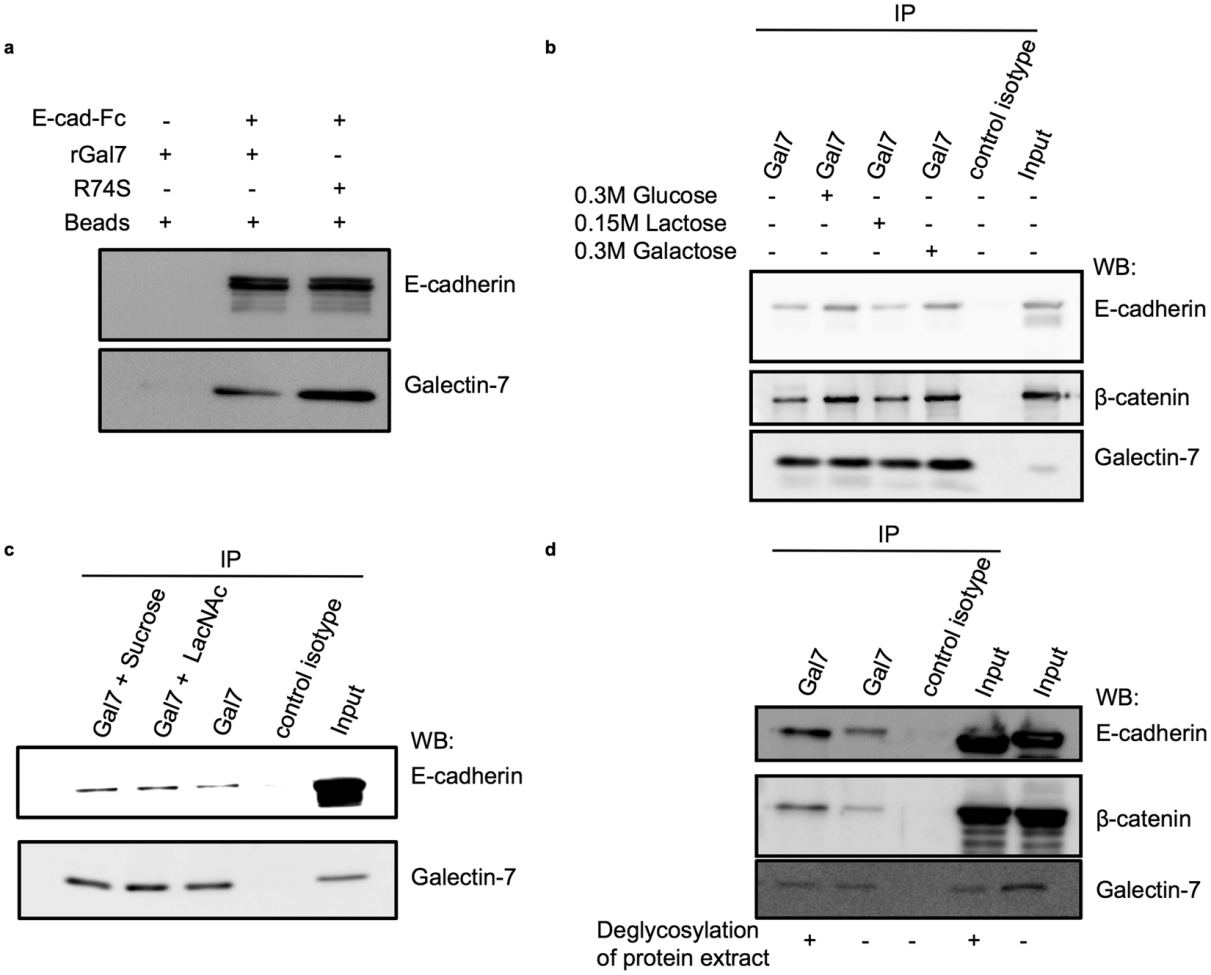
Galectin-7 directly interacts with the extracellular domain of E-cadherin independently of glycosylation motifs. (**a**) *In vitro* binding assays were performed using recombinant wild type human galectin-7 (rGal7); CRD mutated human galectin-7 (R74S) and extracellular domain of mouse E-cadherin fused to human IgG1 Fc fragment (E-cad-Fc). Both WT and mutated galectin-7 (rGal7 and R74S) were precipitated with E-cad-Fc. (**b**) Immunoblot showing co-immunoprecipitations of galectin-7 performed on cell lysates in the presence of high sugar concentrations. (**c**) As in absence of competitive carbohydrate or in the presence of 15 mM sucrose, addition of 15 mM LacNAc did not prevent galectin-7 to immunoprecipitate E-cadherin. (**d**) Immunoprecipitations of galectin-7 were done after deglycosylation of protein extracts in non-denaturing conditions. Representative western blot data are shown in this figure.

To further describe the relationship between AJ components and galectin-7, we investigated whether or not E-cadherin was the direct binding partner of galectin-7 by performing *in vitro* binding assays with purified proteins. First, recombinant human galectin-7 (rGal7) was incubated with a recombinant chimeric E-cadherin containing the ectodomain of the E-cadherin protein (from Asp157 to Val709) fused to a Fc fragment (E-cad-Fc). Strikingly, galectin-7 was precipitated by the extracellular domain of E-cadherin (Fig. 3a), demonstrating a direct interaction between these two proteins. Interestingly, mutated galectin-7 with the R74S substitution in the CRD domain also precipitated *in vitro* with recombinant E-cad-Fc (Fig. 3a). This CRD-defective mutant being unable to bind to sugar motifs^40^, it suggests a possible glycosylation-independent interaction between the extracellular domain of E-cadherin and galectin-7.

### Glycosylation motifs are not mandatory for galectin-7 interaction with E-cadherin and β-catenin

Galectins are generally described as β-galactoside-binding proteins but they can also establish direct protein-protein interactions^41^. In this context, we explored the importance of glycosylation motifs for the interaction of galectin-7 with AJ proteins. Indeed, E-cadherin is a transmembrane glycosylated protein and β-catenin has been reported to be O-GlcNAc (O-linked N-acetylglucosamine) glycosylated^42,43^.

Hence, we performed immunoprecipitations of galectin-7 in the presence of an excess of sugars with a high affinity for galectins (0.15 M lactose or 0.3 M galactose) or in the presence of 0.3 M glucose that does not bind to galectins. The addition of lactose or galactose failed to displace the interaction of galectin-7 with E-cadherin and β-catenin (Fig. 3b). Together with these observations, addition of 15 mM LacNAc, a molecule with a high affinity for galectins, was also insufficient to disrupt galectin-7 and E-cadherin interaction (Fig. 3c).

To strengthen these observations, we submitted the cell lysates to a mixture of deglycosylation enzymes so as to completely remove glycosylation motifs from E-cadherin before immunoprecipitation of galectin-7. Deglycosylation of the protein extracts must be achieved in non-denaturing conditions to preserve the antigenic integrity of the samples for immunoprecipitation. To monitor deglycosylation efficiency of E-cadherin in non-denaturing conditions, we compared the shift of E-cadherin molecular weight in SDS-PAGE after deglycosylation performed with or without denaturing conditions to that of the non-deglycosylated native sample. In both cases, deglycosylation resulted in a similar shift in the apparent molecular size of E-cadherin (supplementary Fig. 2d). Interestingly, deglycosylation of the cell lysate did not prevent galectin-7 to precipitate E-cadherin and β-catenin (Fig. 3d). Taken together, these results demonstrate that galectin-7 interacts with AJ components in a glycosylation-independent manner.

### Galectin-7 is dispensable for correct AJ localisation and assembly at the plasma membrane

**Figure 4:**
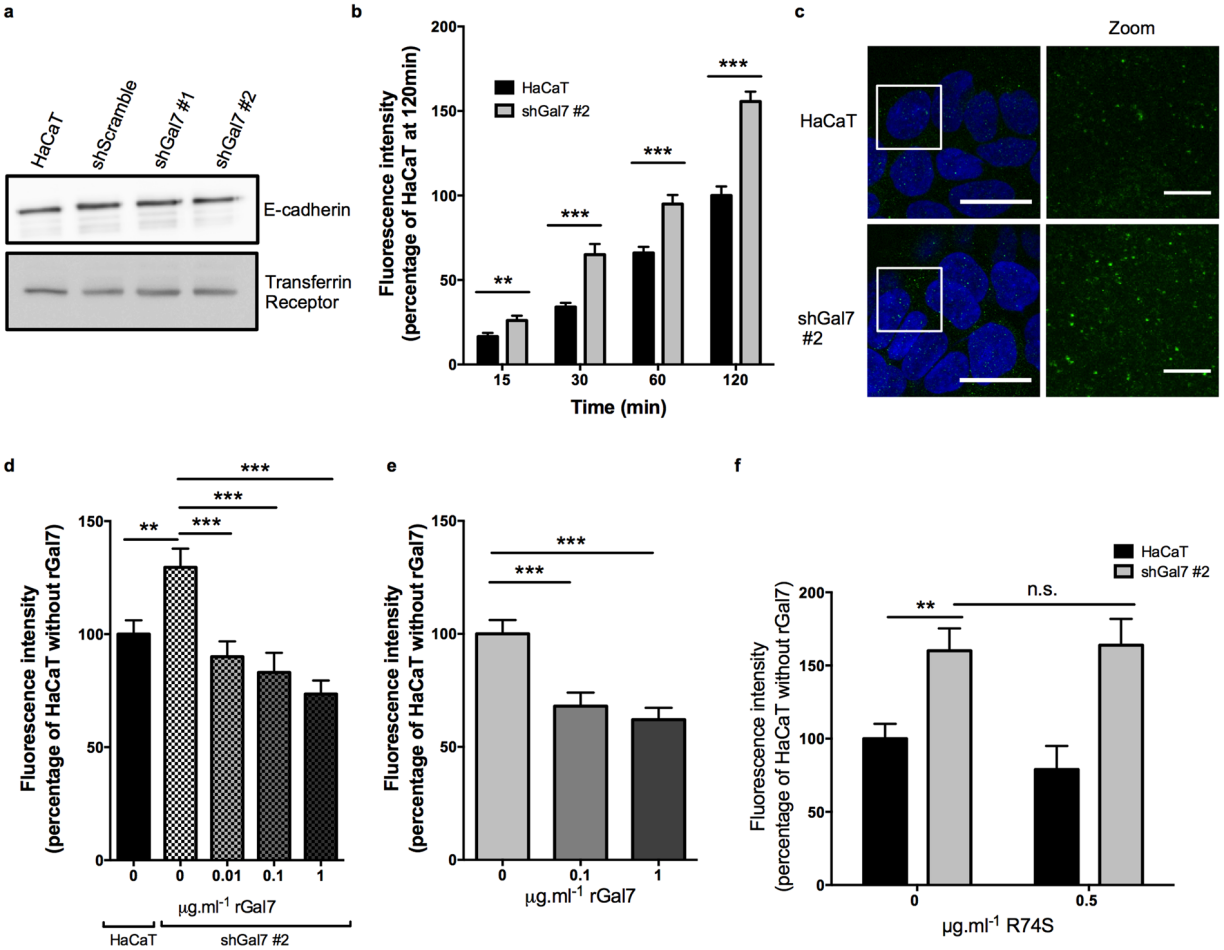
Galectin-7 impacts E-cadherin dynamics at the plasma membrane. (**a**) Total cell surface proteins were purified using labelling with biotin and pull down via avidin affinity. Immunoblots show similar levels of surface E-cadherin in HacaT cells or in shGal7 clones. Transferrin receptor was used as a loading control. (**b**) Quantification of internalized E-cadherin in HaCaT or shGal7 #2 cells at 15, 30, 60 or 120 min after addition of HECD-1 antibody. (**c**) Representative images of internalized E-cadherin after 5 min incubation with HECD-1 antibody, directed against E-cadherin ectodomain. Maximum intensity projection of Z-stacks (Z-steps = 0.21 μm) obtained with ImageJ. Scale bar = 10μm (left panels) or 5μm (zoom, right panels). (**d**,**e**) Quantification of internalized E-cadherin during 30 min at 37 °C after addition of 0 to 1 μg.ml^−1^ recombinant galectin-7 (rGal7) in shGal7 #2 clone (**d**) or control HaCaT (**e**). (**f**) Quantification of internalized E-cadherin in HaCaT or shGal7 #2 cells during 30 min at 37 °C after addition of 0 or 0.5 μg.ml^−1^ recombinant CRD-mutated galectin-7 (R74S). (**b**,**d**,**e**,**f**) Between 15 and 17 measures per condition were performed in n = 3 independent experiments. Mean values ± s.e.m. are represented. P-values obtained from unpaired t-tests with Welch’s correction are represented. ***p < 0.001; **p < 0.01; *p < 0.05.

After identifying an interaction of galectin-7 with AJ proteins, we examined if galectin-7 could influence the establishment of this adhesion complex. In shGal7 clones depleted of galectin-7, we observed by immunofluorescence that E-cadherin (supplementary Fig. S3a), β-catenin (supplementary Fig. S3b) and α-catenin (supplementary Fig. S3c) were correctly recruited to cell-cell contacts indicating that AJ complexes did still assemble properly at the plasma membrane. In addition, the total protein level of E-cadherin was also similar as assessed by flow cytometry (supplementary Fig. S3d). Accordingly, E-cadherin mRNA expression was not modified after galectin-7 knockdown (supplementary Fig. S3e). Importantly, the amount of cell surface E-cadherin, assessed by a cell surface biotinylation assay in confluent HaCaT cells, was also not different after galectin-7 depletion (Fig. 4a).

### Galectin-7 depletion alters E-cadherin dynamics at the plasma membrane

Considering above results, we then explored if galectin-7 could influence the behaviour of the membrane fraction of E-cadherin. Indeed, E-cadherin dynamics is a crucial parameter for AJ integrity. We therefore investigated if galectin-7 depletion may affect E-cadherin turnover at the plasma membrane. For this purpose, we performed an endocytosis assay using anti-E-cadherin HECD-1 antibody specific for the ectodomain of E-cadherin. Strikingly, E-cadherin uptake was significantly increased after galectin-7 depletion compared to control HaCaT cells at the different time points tested (Fig. 4b). This difference was observed after only 5 min of incubation with the presence of a higher amount of endocytosed E-cadherin-containing vesicles in the shGal7 #2 clone compared to control HaCaT cells (Fig. 4c).

To confirm that the modification of E-cadherin dynamics in the shGal7 #2 clone was induced by galectin-7 knockdown, we performed a rescue experiment by adding exogenous rGal7 to the cell media with HECD-1 antibodies in shGal7 #2 clone for 30 min. Addition of 0.01 to 0.1 μg/ml rGal7 to shGal7 #2 clone restored the E-cadherin internalisation levels to that of the reference wild type HaCaT cells (Fig. 4d). Strikingly, addition of a higher dose of rGal7 significantly reduced the amount of endocytosed E-cadherin (Fig. 4d), suggesting that the regulation of E-cadherin endocytosis by galectin-7 is dose-dependent.

Accordingly, addition of 0.1 or 1 μg/ml rGal7 to wild-type HaCaT cells significantly reduced the amount of internalized E-cadherin (Fig. 4e), confirming the involvement of galectin-7 in the regulation of the turnover of E-cadherin at the plasma membrane.

Altogether, these experiments attest that galectin-7 controls E-cadherin endocytosis at the cell surface. Unexpectedly, addition of the mutated R74S galectin-7 was inefficient in restoring normal levels of internalized E-cadherin in galectin-7-depleted cells (Fig. 4f), suggesting that even though the sugar binding activity of galectin-7 is dispensable for its binding to E-cadherin, it is nevertheless required for its E-cadherin membrane retention function.

Finally, we confirmed that the impact of galectin-7 on E-cadherin uptake was antibody-independent by using a cell surface biotinylation approach to monitor membrane proteins internalisation. Indeed, shGal7 #2 clone displayed increased amount of endocytosed biotinylated E-cadherin compared to control HacaT cells (Supplementary Fig. S4). The membrane retention effect of galectin-7 was specific of E-cadherin as endocytosis of the transferrin receptor, another basolateral transmembrane protein, was not affected by galectin-7 knockdown (Supplementary Fig. S4).

### Galectin-7 influences E-cadherin exocytosis

**Figure 5:**
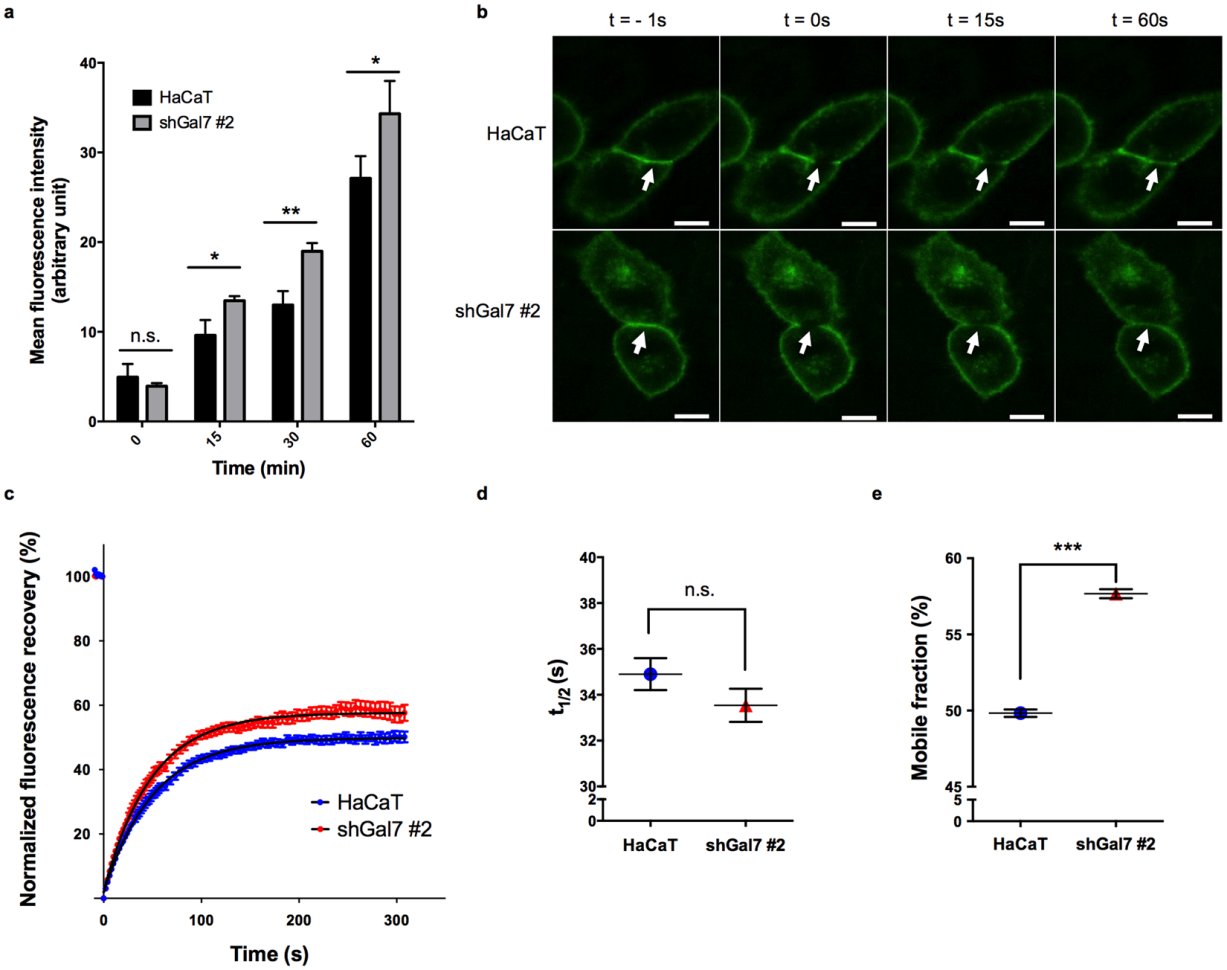
Galectin-7 stabilizes E-cadherin at the plasma membrane in mature adherens junctions. (**a**) Mean surface fluorescence of E-cadherin measured by FACS in non-permeabilized cells. Cells were detached from the plate using trypsin and incubated for the indicated time period at 37 °C to let E-cadherin be re-addressed to the plasma membrane. Mean values ± s.d. are represented (n = 3). (**b**) Representative images of E-cadherin-GFP signal acquired during FRAP experiments in HaCaT cells (upper panel) and in shGal7 #2 clone (lower panel). Arrows indicate the bleach area. Scale bar = 10 μm. (**c**) Normalized fitted FRAP curves (dark) and mean of experimental data (red and blue) showing E-cadherin-GFP recovery after photobleaching. Mean values ± s.e.m are represented (n ≥ 39). (**d**,**e**) Graphs showing halftime recovery (t_1/2_) (**d**) and mobile fraction (**e**) extracted from one phase association fitting curves. Mean values ± s.e.m are represented.

To further explore the membrane turnover of E-cadherin, we then investigated if galectin-7 also influences E-cadherin exocytosis. To assess the rate of surface re-expression of E-cadherin after membrane stripping, we measured by flow cytometry the fluorescence intensity of surface E-cadherin staining after trypsin-induced cell detachment and subsequent incubation at 37 °C. At the different time points tested, galectin-7-depleted cells presented a higher amount of surface E-cadherin (Fig. 5a) whereas the total amount of E-cadherin per cell was equivalent as measured by flow cytometry analysis after cell permeabilisation (Supplementary Fig. 3c). This result indicates that galectin-7 additionally impacts the exocytosis of E-cadherin. Consequently, galectin-7-depleted cells exhibit a similar amount of surface E-cadherin (Fig. 4a) despite increased endocytosis (Fig. 4b) probably due to compensatory enhanced exocytosis (Fig. 5a).

### Galectin-7 stabilizes junctional E-cadherin in mature AJs

We then aimed at determining if galectin-7 regulates the pool of E-cadherin engaged in AJs. As a consequence, we studied the dynamics of trans-interacting E-cadherin at cell-cell contacts by performing FRAP (Fluorescent Recovery After Photobleaching) experiments on mature AJs in nearly confluent monolayers. After photobleaching of a region of mature AJs in transiently expressing E-cadherin-GFP cells, we measured the recovery of fluorescence (Fig. 5c). Interestingly, galectin-7-depleted cells displayed a similar half time recovery (Fig. 5d) but exhibited a significantly increased mobile fraction of E-cadherin compared to control HaCaT cells (Fig. 5e), indicating that the amount of resident membrane E-cadherin in AJs was decreased by galectin-7 knockdown. These results point out that galectin-7 stabilizes junctional E-cadherin at the plasma membrane.

### rGal7 accumulates with E-cadherin at the plasma membrane but is not internalized with E-cadherin

**Figure 6:**
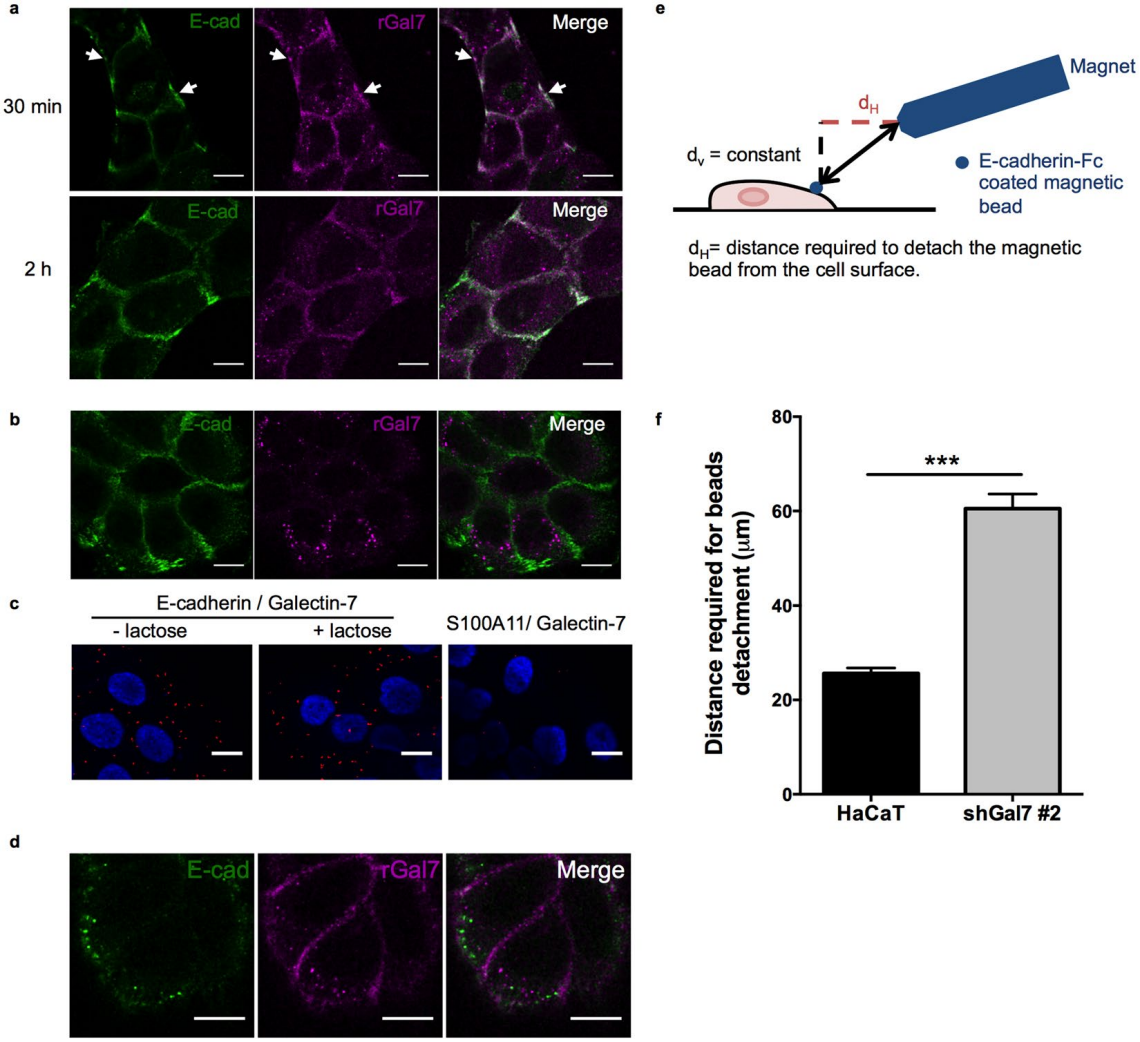
Association of galectin-7 with E-cadherin at the plasma membrane influences E-cadherin mediated adhesion strength. (**a**) Immunofluorescence images showing accumulation of rGal7-Cy3 (magenta) at the plasma membrane with E-cadherin (green) 30 min or 2 h after addition of 0.5 mg.ml^−1^ rGal7. Scale bar = 20 μm. (**b**) rGal-Cy3 enrichment at the plasma membrane was removed by 200 mM lactose washes. Scale bar = 20 μm. (**c**) Washing with lactose gave similar PLA staining than no washing for E-cadherin-Galectin-7 proteins. S100A1 – Galectin-7 staining served as a negative control. Scale bar = 15 μm. (**d**) rGal7-Cy3 (magenta) did not show important colocalisation with endocytosed E-cadherin (green). HECD-1 antibody was incubated 30 min à 37 °C to label endocytosed E-cadherin. Scale bar = 20 μm. (**e**) Schematic representation of the experimental design illustrating E-cadherin coated bead bound to HaCaT cells. Rapprochement of the magnetic tweezers will cause bead detachment. (**f**) Measurements of the distance required to detached E-cadherin coated beads from cells. Mean values ± s.e.m are represented.

To better understand how galectin-7 might influence AJ dynamics, we added exogenous fluorescently labelled rGal7 and observed its subsequent localisation using the intermediate concentration of 0.5 μg.ml^−1^. When rGal7-Cy3 was added to the medium, it accumulated at the plasma membrane 30 min after addition and was still visible at the membrane after 2 h (Fig. 6a). Interestingly, membrane regions with strong enrichment of rGal7-Cy3 exhibited increased staining of E-cadherin (Fig. 6a, arrows). Washing with β-lactose removed rGal7-Cy3 enrichment at the plasma membrane (Fig. 6b), indicating that most of rGal7 binding to the plasma membrane was outside the cells and through the lectin activity. However, when performing PLA experiments after lactose washes in HaCaT cells, similar levels of PLA staining with or without washing steps were retrieved (Fig. 6c), indicating that even if the majority of galectin-7 is washed out, a subpopulation of galectin-7 proteins bound to E-cadherin remained after lactose washes.

Importantly, recombinant galectin-7 was scarcely found in endocytosed E-cadherin-containing vesicles (Fig. 6d), indicating that galectin-7 was mainly not endocytosed with E-cadherin and did not travel with it. These results suggest that galectin-7 probably influences the fate of E-cadherin at the plasma membrane but not in vesicular compartments.

### Galectin-7 depletion reduces E-cadherin-mediated adhesion

To test whether the changes in E-cadherin dynamics due to galectin-7 depletion had a functional impact on AJ-based intercellular adhesion, we measured the strength of trans-association of E-cadherin using magnetic bead experiments. In this assay, cells were incubated with E-cadherin-coated magnetic beads for 30 min. Then, we evaluated the adhesion strength by measuring the distance required to detach the beads from the cell surface with a magnetic tweezers (Fig. 6e). Indeed, the bead is submitted to a force proportional to the local magnetic field gradient. As a consequence, the force increases with the decrease of the distance from the magnetic tweezers^15,44^. Interestingly, this distance was higher after galectin-7 depletion, indicating that the strength required to detach the beads was smaller and thus, that AJ adhesion strength was weakened by galectin-7 silencing (Fig. 6f). In conclusion, the modification of E-cadherin dynamics at the plasma membrane induced by galectin-7 downregulation that we demonstrated above is correlated with a decrease of E-cadherin-dependent adhesion.

## Discussion

The present study provides the first evidence that galectin-7 regulates intercellular interactions favouring coordinated and efficient collective cell migration. We propose that this regulation is through modulation of E-cadherin dynamics at the plasma membrane. Indeed, we show that galectin-7 directly binds to the extracellular domain of E-cadherin and stabilizes it at the plasma membrane. This surface retention of E-cadherin mediated by galectin-7 requires the carbohydrate binding activity of galectin-7 even if a functional CRD domain of galectin-7 is not mandatory for interaction with E-cadherin.

Galectin-7 has been previously implicated in cell migration during re-epithelialisation events after corneal^30^ or epidermal injury^31,32^. Moreover, this lectin has been shown to promote invasiveness during cancer progression^45–47^. In the present study, by focusing more precisely on epithelial cell migrating behaviour, we specified the role of galectin-7 and highlighted a previously undescribed function of galectin-7 in the control of the coordination of migrating keratinocytes. Indeed, galectin-7 depletion, in addition to reducing the cell speed in migrating monolayers, induces disorganized and mis-oriented migration. Consistently, we identified galectin-7 as a direct partner of the AJ protein E-cadherin, providing a rational explanation for the observed phenotype. Indeed, E-cadherin has been shown to regulate intercellular coordination during collective migration^6,10,11,48^. During this process, AJs need to be constantly renewed and redistributed at the cell level between migrating neighbouring cells^49,50^. Correct interactions and communications between leader cells, at the front of the free edge, and follower cells dictate the behaviour of individual cells to preserve the integrity of the migrating sheet and to prevent random migration^4^. As an illustration, re-distribution of AJ components during collective cell migration determines localisation of focal adhesion and subsequent acquisition of the front-to-rear polarity^51–53^. Notably, both coordination defects during migration and altered establishment of the front-to-rear polarity are detected after galectin-7 silencing.

Using varying levels of galectin-7, we demonstrated its implication in the regulation of E-cadherin dynamics at the plasma membrane of keratinocytes. Indeed, whereas silencing of galectin-7 increases E-cadherin internalisation, addition of exogenous galectin-7 stabilises E-cadherin at the plasma membrane. Interestingly, addition of fluorescently labelled exogenous galectin-7 displayed accumulation at cell-cell contacts but was not enriched in vesicles containing endocytosed E-cadherin. Hence, we conclude that galectin-7 is a stabilisation factor of E-cadherin at the plasma membrane but is not trafficking with it inside the cell. Moreover, our results indicate that the enhanced amount of E-cadherin endocytosed in galectin-7-depleted cells is compensated by an increase of exocytosis of the protein, resulting in a similar level of surface E-cadherin but increased turnover rates. E-cadherin dynamics at the plasma membrane is a key parameter controlling AJ plasticity during maintenance or remodelling processes^12,13,18,19^. Notably, AJs are rapidly renewed complexes with the residence time of single E-cadherin molecules in AJ complexes in the order of minutes in numerous cell lines^18,51,54^; however this residence time might be longer in living animals^55^. Studying how E-cadherin dynamics is regulated in different environments or different cell types is essential. Indeed, it is crucial for the cells to adapt E-cadherin turnover to various conditions and galectin-7-mediated stabilisation of E-cadherin could be one of the control mechanisms in pluristratified epithelia. Galectins, through their cell type-specific expression profile, their dynamic behaviour, and their rapid redistribution, are good candidates to control adaptive responses. To illustrate this point, E-cadherin dynamics is likely to adapt to the switch from proliferating to the differentiated state of the cell^20,56^ during epidermal renewing and epidermal differentiation. Interestingly, galectin-7 localisation is strongly enriched in regions of cell-cell contacts with AJ proteins in the upper layers of the epidermis, where cells are going through differentiation and should adapt their AJ dynamics.

Moreover, this study reinforces the link between AJ dynamics and intercellular adhesion strength. Indeed, modification of E-cadherin turnover at the plasma membrane due to galectin-7 silencing is associated with a strong decrease in E-cadherin-mediated AJ strength even if the total amount of E-cadherin at the plasma membrane remains constant. A correct balance between AJ plasticity and stability is essential to maintain intercellular cohesion and communication while favouring an adaptive response to perturbations or variations of cellular conditions^12,15,18,19,57^. In particular during migration, intercellular adhesion strength needs to be adapted to allow cell displacement while preserving information exchange to prevent less efficient random migration^58^. We propose that during collective migration of epithelial cells, a specific amount of galectin-7 mediates the stabilisation of E-cadherin at the plasma membrane, generating sufficient adhesion strength and allowing correct communication and force transmission for proper collective migration. This model predicts that decreased levels of galectin-7 should reduce the stability of AJ complexes and thus intercellular coordination during migration whereas conversely, excess of galectin-7 should reduce E-cadherin internalisation and thus decrease cell motility^49^ by increasing adhesion. Accordingly, our group previously reported that both overexpression of galectin-7^32^ or absence of galectin-7^31^ delayed wound healing in mice, highlighting the importance of a correct balance between stability and dynamics.

Strikingly, this work demonstrated that galectin-7 is able to directly bind to the extracellular domain of E-cadherin but this interaction is not through glycosylation motifs. Galectin-7 had already been shown in one case to form direct protein-protein interaction with Bcl-2 protein in mitochondria^59^. Here, we reveal a new example of this lectin associating with proteins independently of carbohydrate groups. Indeed, not only deglycosylation of protein extracts or competition experiments with carbohydrates failed to prevent galectin-7 from precipitating E-cadherin, but also the CRD-defective mutant galectin-7 (R74S)^40^, was able to bind *in vitro* to the extracellular domain of E-cadherin. However, the sugar binding activity of galectin-7 seems to be required for its stabilisation of surface E-cadherin since mutant galectin-7 R74S is unable to restore proper E-cadherin dynamics. These results suggest the involvement of an additional glycosylated protein or glycolipid. Knowing that galectins can form bridges between membrane molecules, such as in the formation of lattice domains^60^, we propose that galectin-7 could mediate E-cadherin association with a glycosylated molecule, glycosylated E-cadherin itself or another protein, and this interaction would stabilise E-cadherin at the plasma membrane. This model implies a poorly described role for the ectodomain of E-cadherin in the regulation of its turnover. Nevertheless, this domain is already known to initiate cis-association of E-cadherin molecules^14^ and subsequent anchoring to the cytoskeleton and AJ stabilisation^15^. An illustration of the implication of the E-cadherin extracellular domain has also been shown in cancer where deletions in the ectodomain modify E-cadherin endocytosis^61^ but the molecular basis remains unknown.

E-cadherin cellular level or E-cadherin dynamics at the plasma membrane have been described to be central in cancer biology and especially in tumor dissemination^62,63^. In this study, only by adding exogenous recombinant galectin-7 directly to the medium of the cells, we managed to control galectin-7 function at AJs, inducing increased retention of E-cadherin at the plasma membrane. Consequently, galectin-7 appears as a useful therapeutic tool to regulate AJ dynamics and delay cancer progression. Because galectins have been recognized as modulators of tumor progression^64^, efforts have been made to generate galectin-targeting compounds^65,66^ and several molecules have entered clinical trials^67^. Their implication on the regulation of AJ dynamics and intercellular adhesion should thus be considered.

## Methods

### Cell culture

The HaCaT cell line (Human adult low Calcium high Temperature) were grown in Dulbecco’s Modified Eagle Media (DMEM, Invitrogen) supplemented with 2 mM L-glutamine (Invitrogen), 10 units.ml^−1^ penicillin, 10 μg.ml^−1^ streptomycin (Invitrogen) and 10% foetal bovine serum (FBS) in a5% CO_2_ atmosphere at 37 °C. Two independent clones with reduced expression of galectin-7 were generated by stable transfection of two shRNAs (sequence shGal7 #1: 5′-CCGGGCTGAGAATTCGCGGCTTGGTCTCGAGCCAAGCCGCGAATTCTCAGCTTTTTG-3′; sequence shGal7 #2: 5′-CCGGCTCATCATCGCGTCAGACGACTCGAGCGTCTGACGCGATGATGAGCTTTTTG-3′) inserted in a plasmid carrying a pLOK.1 selection cassette for puromycin (TRCN0000057395 and TRCN0000057396, Sigma-Aldrich). The clones having integrated the shRNA directed against galectin-7 mRNA were selected with 2 μg.ml^−1^ puromycin. A control clone was generated by infecting HaCaT cells with the pLKO.1-puro non-target shRNA Control Plasmid (SHC016, Sigma-Aldrich).

### Animals

Mice were kept on a C57Bl/6 background and housed in a specific pathogen-free animal facility. All experiments were performed on 2 month-old female mice. Animals were handled respecting the French regulations for animal care, and the Animal Experimentation Ethical Committee Buffon (CEEA-40) approved all mice work.

### Histology and immunostaining

Tissue processing and immunostaining were performed as previously described^32^. The following primary antibodies were used: rabbit polyclonal anti-galectin-7 antibodies (ab10482, Abcam) and mouse monoclonal anti-β-catenin antibody (MA1-301, Thermo Scientific).

Nuclei were stained with Hoechst33342 (H3570, Invitrogen) and confocal acquisition was performed using a Leica SP5 microscope. Pearson’s r coefficient was measured using JACoP plugin from ImageJ on a selection including all the layers of the epidermis.

### Antibody uptake experiments

Cells were incubated in fresh growth media containing the anti-E-cadherin HECD-1 antibody (1:500, ab1416, Abcam) on ice or at 37 °C for different periods of time. Surface-bound antibodies were removed by 3 × 5 min acid washes (0.5 M acetic acid, 0.5 M NaCl in PBS) under agitation on ice. Cells were washed with ice-cold PBS++ (PBS+ 1 mM CaCl_2_ + 0.5 mM MgCl_2_), then fixed in 4% paraformaldehyde for 20 min at room temperature and processed for immunofluorescence. The images for quantification were taken with a DMRA2 Leica Microscope. For quantification, the images were background subtracted and cellular regions were identified to measure the total fluorescence intensity using the ImageJ software. Fluorescence intensity measured from cells incubated with HECD-1 antibody 1 h at 4 °C was removed from the values measured for the corresponding clones. For each condition tested, three independents experiments were performed and around 15 images were analysed per experiment.

### Fluorescence Recovery After Photobleaching

Cells were imaged 48 h after transient transfection with a human E-Cadherin-GFP construct. During acquisition, the cells were maintained at 37 °C in a 5% CO_2_ chamber in a serum-free medium. Images of 256 × 256 pixels were taken with a 63X immersion objective in a confocal microscope (SP5, Leica). A rectangular region of interest at cell-cell junctions was chosen and cells were imaged with 9% 488 nm laser power as follow: 7 reference images every 1.3 s, 50 post-bleached images every 1.3 s and 50 post-bleached images every 5 s. Efficient photo-bleaching was achieved using 100% 488 nm laser power and a one frame bleach time. At least 39 sequences were acquired for each condition.

### Data Analysis of FRAP experiments

Using ImageJ software, three areas were quantified for mean grey values: the bleached area (1), a non-bleached area at cell-cell contact (2) and a background area outside the cells (3). Image intensities of area (1) and (2) were background subtracted and intensities of (1) corrected for the bleaching effect due to time-lapse acquisition using the bleaching coefficient measured with the area (2). Fluorescence values from area (1) were then normalized between 0 and 100%. Mobile fraction and half time recovery parameters were extracted from the one phase association equation I_t_ = I_0_ + (I_∞_ − I_0_) × (1 − e^−t/τ^) where I is the intensity measured, t the time and τ the time constant) fitted with experimental data using the Prism 6.0 software (Graphpad).

### *In vitro* wound healing assay

Cells were plated in 12-well plates on both sides of a plexiglass insert that was removed once the cells had reached confluence (T0). Cells in the entire well were imaged at T0 and T16 (T = 16 h) and the wound closure was calculated by the difference of the covered area by the cell monolayer between T0 and T16. Images were taken with a Leica MZFLIII system through an Axiocam HRc from Zeiss. Results are mean of three independent experiments performed in triplicate.

Videomicroscopy of migrating HaCaT cells on a fibronectin matrix was performed on a Biostation (Nikon) during 24 h after PDMS insert removal. Cell trajectories were manually obtained using the MTrackJ ImageJ plugin. Cell speed was calculated by a regression analysis of the plot representing the distance versus the time. Directionality was calculated as cosθ where θ is the angle between the field vector at t = 18 h and the cell migration direction; the persistence was calculated after 18 h as the ratio of the cumulative distance over the Euclidian distance. Velocity correlation length was measured as described previously^15^. The separation time was estimated manually by following two cells that were neighbours at t = 0 h and measuring the time required for the cells to separate from each other.

### Immunofluorescence

Cells were washed with 1x PBS and fixed for 20 min in paraformaldehyde (PFA) 4% at room temperature. After two washes with 1x PBS, they were permeabilized for 20 min in 1x PBS - 0.025% saponin and then blocked for 30 min in 1x PBS - 0.025% Saponin - 1% BSA (Bovine Serum Albumin, Sigma-Aldrich). Cells are incubated overnight at 4 °C in 1x PBS - 0.025% saponin, 1% BSA containing the primary antibody. The following day, after washing with 1x PBS, cells were incubated in 1x PBS - 0.025% saponin - 1% BSA containing the secondary antibody coupled to a fluorochrome for 1 h protected from light at room temperature. After two washes with 1x PBS, the nuclei were stained with 10 μg.ml^−1^ Hoechst33342 (H357C, Invitrogen) for 20 min. Slides were washed with 1x PBS, then with distilled water before being mounted on slides with Fluoromouont-G (0100-01, SouthernBiotech). The slides were visualized using an SP5 confocal scanning Tandem RS (Leica) and analyzed by ImageJ. The following antibodies were used: anti-γ-tubulin (T6557, Sigma-Aldrich); anti-GM130 (610823, BD Biosciences); anti-E-cadherin (ab1416, Abcam); anti-E-cadherin (610181, BD Biosciences); anti-Galectin-7 (ab10482, Abcam); anti-β-catenin (MA1-301, Thermo Scientific); anti-α-catenin (13-9700, Thermo Scientific); anti-α-catenin (ab51032, Abcam); anti-S100A11 (ab180593, Abcam).

### Flow Cytometry

5 × 10^5^ cells were prepared for each condition. They were trypsinized, then washed and incubated for 15 min, 30 min or 1 h with growth medium supplemented with 10% FBS at 37 °C. Following this step, the cells were washed with PBS/FBS 2% and fixed with PFA 2% for 20 min at 4 °C. They were then washed with PBS and incubated with cold FBS for 15 min at 4 °C before addition for 1 h at 4 °C of the anti-E-cadherin primary antibody recognizing the ectodomain of E-cadherin (#3195 S, Cell Signalling) diluted in PBS/FBS 2%. The cells were washed twice and the secondary antibody was added for 30 min at room temperature (RT) in the dark. They were then washed 3 times and re-suspended in 500 μl PBS for analysis on a CyAn ADP 9 C analyser (Beckman Coulter). At least 10^4^ cells were analysed per condition (n = 3).

### Co-immunoprecipitation

Experiments were performed as previously described^32^.

Whole cell extracts were prepared from confluent cells grown on 10 cm tissue culture dishes. For competition experiments with sugar, all steps were performed with either 150 mM β-lactose, 300 mM galactose or 300 mM glucose. For competition experiments with 15 mM LacNac (N-Acetyl-lactosamine, Sigma) or 15 mM sucrose, two additional washing steps with the carbohydrate were performed.

### *In vitro* binding assay

0.3 M of purified proteins were mixed and incubated in 100 μl of lysis buffer overnight at 4 °C under agitation. Then, 60 μl of protein-A-Sepharose (P9424, Sigma) was added and samples were incubated 3 h at 4 °C under agitation. Samples were washed two times with 1X PBS – 0.5% NP40 and two times with 1X PBS.

### Deglycosylation of protein extract

Glycosylation removal was performed on 100 μg proteins from cell lysates using a Protein Deglycosylation Mix (P6039S, New England Biolabs) containing O-glycosidase, PNGase F, neuraminidase, β1-4 galactosidase and β-N-Acetyl glucosaminidase according to the manufacturer instructions. The proteins were incubated with the enzyme cocktail and incubated 6 h at 37 °C. The deglycosylation status of the protein extract was assessed by mobility shift on SDS-PAGE gels.

### Western Blot

Proteins (30 μg of total cell lysates) were separated in an SDS-PAGE gel and transferred to a PVDF membrane (AmershamHybond-P, GE Healthcare). The membranes were then blocked in PBS-T (1X PBS - 0.1% Tween 20) supplemented with 5% non-fat milk for 1 h at RT and incubated with the primary antibody overnight at 4 °C. Immunoblots were visualized using a horseradish peroxidase-conjugated secondary antibody followed by enhanced chemiluminescence detection with an ImageQuant LAS 4000 developer (GE Healthcare).

### Proximity Ligation Assay

The assay was performed with the Duolink® *In situ* Red Starter kit from Sigma-Aldrich according to the manufacturer’s instructions.

### Magnetic tweezers assay

One μl of 2.8 μm magnetic protein A–coated beads (Dynabeads; Invitrogen) was washed three times in 0.1 M borate buffer, pH 8.0 and resuspended in 100 μl of 1x PBS, before 2 × 30 s sonication. Then, 10 μl of mIgG (#349050, BD Biosciences) were added and left to incubate overnight on a wheel at 4 °C. The next day, beads were incubated with PBS - 1% BSA for 30 min at RT with gentle agitation and washed three times with 1x PBS and resuspended in 200 μl PBS - 1% BSA before 2 × 30 s sonication. Then 1 μl of recombinant mEcad-Fc (748-EC, R&D systems) was added and the mixture left to incubate for 2 h on a rotating wheel at RT. The beads were then washed, resuspended in 200 μl PBS - 1% BSA and sonicated 2 × 1 s before use. Fifty μl of this Ecad-coated bead solution were added to cells grown on a 22 × 22 mm glass coverslip placed on a 3.5 cm Petri dish for 30 min. Extensive washes with culture medium were performed to remove unbound beads. Images were taken with an Olympus IX73 microscope equipped with a 10X objective. Magnetic tweezers assays were then performed as previously described in^15^.

## Electronic supplementary material

Video_HaCaT_WT

Video_shGal7 #2

Supplementary informations
