# Supplementary material for: E-cadherin dynamics is regulated by galectin-7 at epithelial cell surface
Source: Sci Rep. 2017 Dec 6;7:17086. doi: 10.1038/s41598-017-17332-y (PMC5719072; doi:10.1038/s41598-017-17332-y)
Supplement: Supplementary file 3 — Supplementary informations [file 41598_2017_17332_MOESM3_ESM.pdf]

Title
E-cadherin dynamics is regulated by galectin-7 at epithelial cell surface.

Authors
Tamara Advedissian[1], Véronique Proux-Gillardeaux[2], Rachel Nkosi[1], Grégoire Peyret[3], Thao Nguyen[3], Françoise Poirier[1], Mireille Viguier[1*#] and Frédérique Deshayes[1* #].
* co- corresponding authors
# co-seniors.
e-mail address: frederique.deshayes@ijm.fr ; mireille.viguier@univ-paris-diderot.fr

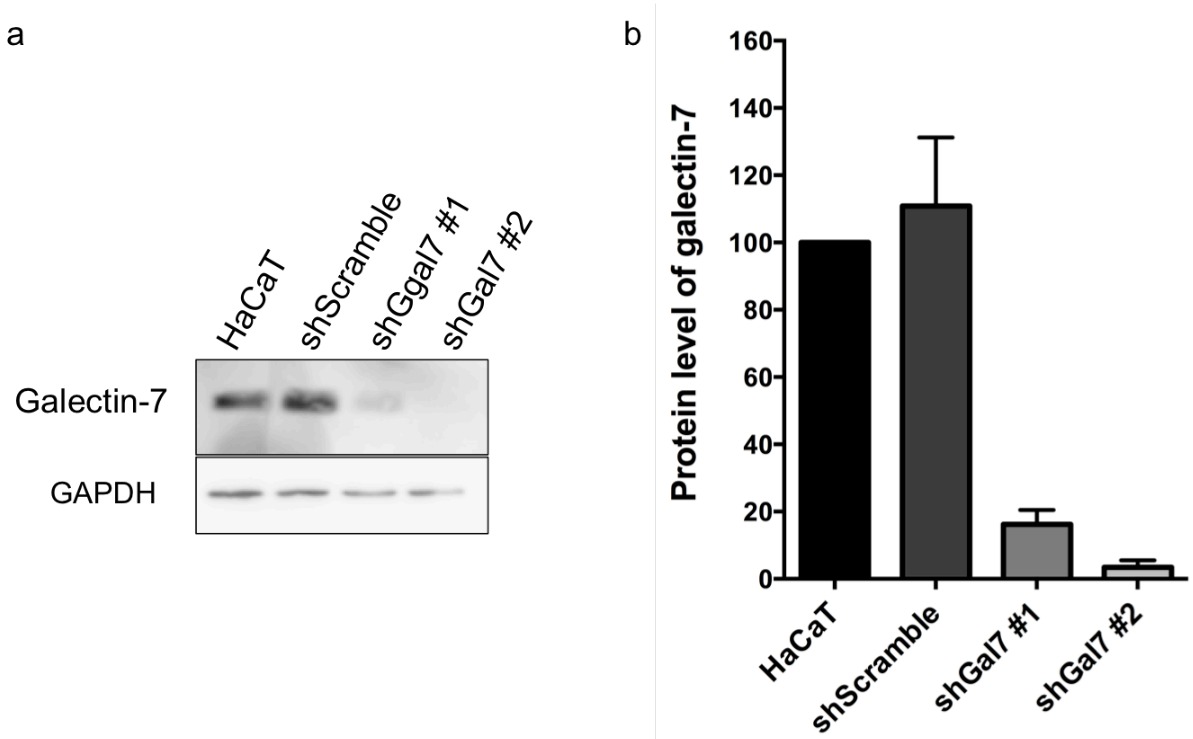

Supplementary Figure 1:
(a) Representative immunoblot showing galectin-7 extinction in shGal7 clones.
(b) Quantification of galectin-7 protein levels from immunoblots. Mean ± s.e.m are represented (n=4).

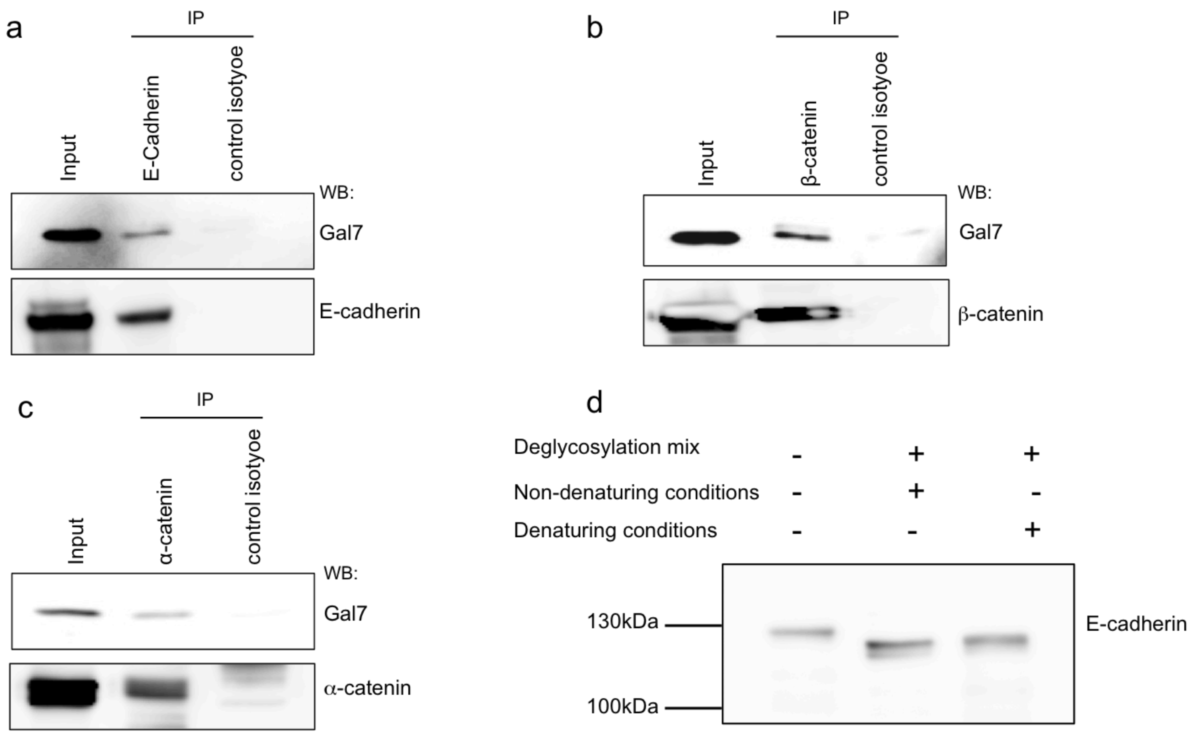

Supplementary Figure 2:
(a, b, c) Western blots showing immunoprecipitation of galectin-7 by E-cadherin (a), β-catenin (b) and α-catenin (c).
(d) Mobility shift of E-cadherin after lysate deglycosylation shows modification of E-cadherin molecular weight.

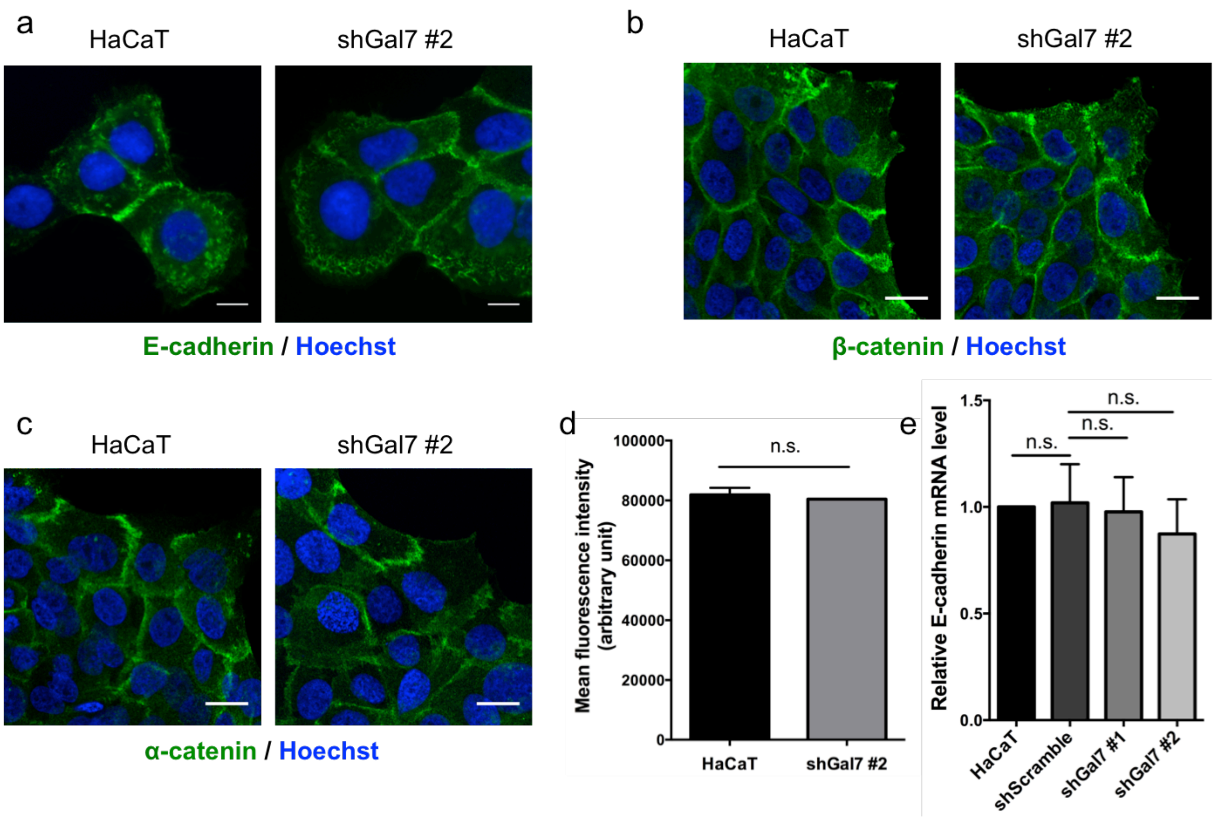

Supplementary Figure 3:
(a) Immunostaining of E-cadherin in HaCaT cells or shGal7 #2 clones shows correct localisation of E-cadherin at cell-cell contacts after galectin-7 depletion. Scale bar = 10 μm.
(b, c) Confocal images of β-catenin (b) and α-catenin (c) in HaCaT cells or shGal7 #2 clones show correct localisation of both catenins at cell-cell contacts after galectin-7 depletion. Scale bar = 20 μm.
(d) Total E-cadherin protein level measured by flow cytometry after cell permeabilization shows similar amount of E-cadherin in HaCaT cells.
(e) Expression level of E-cadherin mRNA measured by qPCR. Mean ± s.e.m are represented (n=3).

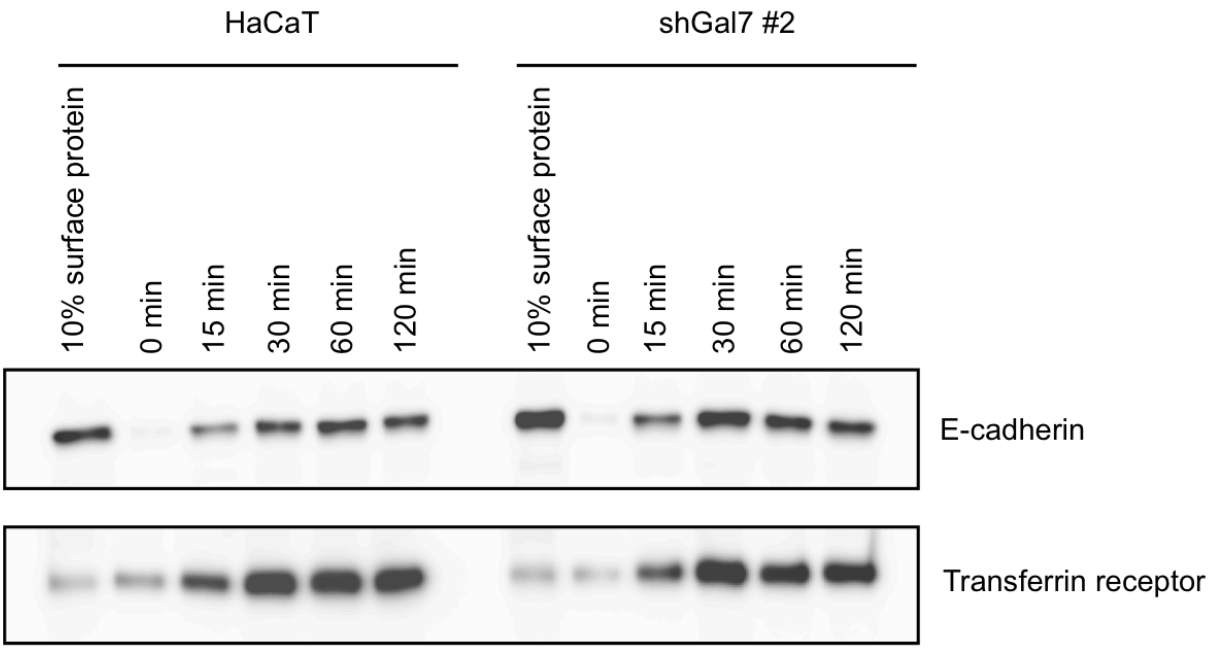

Supplementary figure 4:
Uptake of surface-biotinylated proteins. Cells were surface-biotinylated at 4°C and then incubated at 37°C for 0 to 120 min. After glutathione surface stripping and cell lysis, intracellular biotinylated proteins were captured using NeutrAvidin. Ten percent of total surface proteins were loaded as a control. Immunoblots showed increased endocytosis of E-cadherin but no modification of the transferrin receptor uptake in shGal7 #2 clone compared to control HaCaT cells. Representative images from three independent experiments are shown.

Supplementary Materials

Cell surface biotinylation assay for endocytosis measurement

HaCaT cells were incubated on ice with 0.5 $mg.ml^{-1}$ EZ-link Sulfo-NHS-SS-Biotin (Cat. 21331, ThermoFisher Scientific) in PBS++ for 30 min under agitation. The free biotin was quenched by 2 x 15 min washing of 50 mM NH_4Cl in PBS++ and 2 x 5 min PBS++. Cells were then kept at 4°C or incubated at 37°C in DMEM for 15, 30, 60 or 120 min. All cell surface groups were removed by 2 x 20 min washes on ice using glutathione solution (60 mM glutathione, 75 mM NaCl, 75 mM NaOH, 1% BSA at pH 7.5 - 8.0). Afterwards, cells were washed three times with PBS++ and lysed with RIPA buffer (50mM Tris pH 7.4, 150 mM NaCl, 1mM EDTA, 0.1% SDS, 1% NP40, 0.5% sodium deoxycholate, 1X protease inhibitor cocktail). Next, 65 µl of NeutrAvidin agarose (Cat. 29201, ThermoFisher Scientific) was added to the cell lysates and the mixture was incubated overnight at 4°C under agitation. Cell lysates were centrifuged at 3000g for 3 min at 4°C and washed three time with RIPA buffer. Biotinylated proteins were then eluted using laemmli buffer (Cat. S3401, Sigma-Aldrich) and boiled at 95°C for 5 min before being submitted to SDS-PAGE.
